# Case of "Orbital Aneurism" Treated by Ligature of the Carotid

**Published:** 1890-06

**Authors:** A. W. Prichard

**Affiliations:** Surgeon to the Bristol Royal Infirmary


### CASE OF "ORBITAL ANEURISM" TREATED BY LIGATURE OF THE CAROTID. By A. W. PRICHARD, M.R.C.S.; Surgeon to the Bristol Royal Infirmary.

In 1887 I recorded in the *Bristol Medico-Chirurgical Journal* a case of aneurism of the orbit, in which the symptoms were very much ameliorated by ligature of the carotid. That patient is still alive, and suffers very little inconvenience from the slight noise which he still hears in his head, and which sound is easily audible to anyone applying a stethoscope to the temporal region on the affected side. I now bring forward another case.

J. M., a short, spare man of 30, but who looks considerably older, came under my care last November with the following history: On September 10th last, while at work at Mr. Christopher Thomas' soap works, he fell from a stool and struck the back of his head. The fall stunned him, but he quickly came round; and although a small lump appeared behind the left ear, he was able to continue at work for the next two weeks. The small swelling went away with bathing; but a pain persisted. Fifteen days after the fall he felt something snap just external to his left eye, and a whizzing noise began in his left ear. This noise, he says, gradually increased in intensity. He became very unwell just after the sound commenced, and had to stay in bed, complaining of weakness and nervousness, loss of appetite, and considerable pain in the left side of the head, which was relieved by poultices.

He presented himself at the Bristol Eye Dispensary on November 9th, complaining of double vision. There was slight prominence of the left eyeball, and a difficulty in moving it; some swelling of the lids, and a good deal of conjunctival irritation. The retinal veins were distinctly full in comparison with those of the right eye, and there was a very slight pulsation of the globe. He suffered pain all along the left side of the head; and a bruit could be heard on the left side of the head and on the eye, this bruit then being loudest over the base of the mastoid.

He was given 10 gr. of iodide of potassium three times a day.

For two months I kept him under observation; and although the pain and annoyance to the patient of the sound in his head lessened, still the proptosis increased, and the bruit and pulsation became more marked, and a distinct thrill could be felt in the upper eyelid, and he became somewhat deaf in his left ear. Under these circumstances, I took him into the Infirmary, with a view to further proceedings.

On admission he was found to have good vision in both eyes; the movements of the eyeball tolerably free, but not in accordance with those of the other; the eyeball was advanced $\frac{3}{8}$ of an inch, and the conjunctiva inflamed.

The veins of the left side of the face and temple were fuller than those of the right. The pupils were of the same size; but the movements of the iris sluggish on the affected side. The bruit was very loud, and heard all over the head; louder on the left side, and loudest at a point on the forehead immediately over the centre of the orbit. It was generally louder over the sinuses of the dura mater than on other parts of the skull. The bruit quite disappeared when the carotid artery on that side was compressed. There was no nerve-symptom. In the heart a presystolic murmur could be heard; the radial pulses were unequal in strength and time, the left being weaker. There was no abnormal temperature, and nothing wrong in the lungs, kidneys, or digestive organs.

As the patient was very much troubled by the unpleasant noise in his head, and urgent that something should be tried, and as the condition was certainly growing worse, on June 21st, after the usual consultation, I tied the left common carotid.

The wound healed very well, and the effect of the operation was in some respects satisfactory. The bruit returned in the evening after the operation, but lessened in a few days, and now, six weeks later, is very limited in its loudness and area, compared with what it was before. Moreover, the patient does not hear it himself: the deafness has gone; the protrusion of the globe is not so marked; and the conjunctivitis and the diplopia are both much better. He has occasionally a little pain in the side of the head.

This case was probably one in which a direct communication occurred between the carotid artery and the cavernous sinus. Though stunned by the fall, he was not taken really ill till he felt a snap inside his head a fortnight later; and then the distressing symptom began.

Possibly by fracturing or jarring the petrous portion of the temporal bone or the sphenoid just in front, the fall injured the carotid in or close to the cavernous sinus, and the snap occurred when the injured vessel gave way. The stream of arterial blood poured into the sinus would greatly interfere with the return from the large ophthalmic vein, which is almost a direct continuation of the sinus, and so cause engorgement of all veins in and in the neighbourhood of the orbit, and œdema of the tissues, and so protrusion of the globe. Moreover, the thrill to be felt in the upper lid, and the pulsation, cannot be easily explained in any other way than by the veins receiving an impulse directly through the communication between the artery and vein; and a strong proof that the sound is caused by such a communication lies in the fact that it could be heard most easily over any of the sinuses of the dura mater, and, therefore, has been conducted more by the blood in the sinuses than by the cranial bones. Also, I think that the partial success of the operation helps to prove that this view of the condition of things is the correct one; for by diminishing the amount of blood sent into the sinus by the artery, the ligature of the carotid ameliorated all symptoms, but the freedom of the collateral circulation prevented the wound between the artery and vein from quite closing.

The result is not perfect: but I found a few days ago that by compressing the other carotid, I could quite stop the bruit; and if the patient at any subsequent time gets worse, I shall put before him the question of repeating the operation on the other side.

On May 15th, when this paper was in the printer's hands, I received information of the death of my patient.

He was at work on the morning of May 15th, and came home at dinner-time, and died suddenly.

Next day I made a *post mortem* examination, assisted by Mr. Ormerod and Mr. Pitt, and found both lateral ventricles and the fourth ventricle full of blood; this was doubtless the cause of death.

On examining the middle fossa of the base of the skull, we found an united fracture of the petrous portion of the left temporal bone running up towards the cavernous sinus. On passing a probe down the artery where it had been cut from the circle of Willis in removing the brain, we could see the probe in the artery through two rounded holes in the wall of the sinus.

The preparation is in the museum of the Infirmary.

